# Strain-dependent resistance of yellow mealworm (Coleoptera: Tenebrionidae) to bacterial infection with and without probiotic supplementation

**DOI:** 10.1093/jee/toag064

**Published:** 2026-03-22

**Authors:** Guillaume Saint-Jacques, Étienne Normandin, Chloé Losier, Naomi Gunny, Daphné Larivière, Marie-Odile Benoit-Biancamano, Colin Favret

**Affiliations:** Département de Sciences Biologiques, Université de Montréal, Montréal, QC, Canada; Institut de Recherche en Biologie Végétale, Montréal, QC, Canada; Département de Sciences Biologiques, Université de Montréal, Montréal, QC, Canada; Institut de Recherche en Biologie Végétale, Montréal, QC, Canada; Département de Sciences Biologiques, Université de Montréal, Montréal, QC, Canada; Institut de Recherche en Biologie Végétale, Montréal, QC, Canada; Département de Sciences Biologiques, Université de Montréal, Montréal, QC, Canada; Département de Sciences Biologiques, Université de Montréal, Montréal, QC, Canada; Groupe de Recherche sur les Maladies Infectieuses en Production Animale (GREMIP), Centre de Recherche en Infectiologie Porcine et Avicole (CRIPA), Département de Pathologie et Microbiologie, Faculté de Médecine Vétérinaire, Université de Montréal, Saint-Hyacinthe, QC, Canada; Département de Sciences Biologiques, Université de Montréal, Montréal, QC, Canada; Institut de Recherche en Biologie Végétale, Montréal, QC, Canada

**Keywords:** mealworm, strain, probiotics, entomopathogen, survival

## Abstract

*Tenebrio molitor* L. (Coleoptera: Tenebrionidae) larvae are increasingly farmed worldwide as a sustainable source of edible animal protein, but pathogen outbreaks can severely reduce productivity and revenue. As with other forms of agriculture, identifying and rearing naturally disease-resistant strains could reduce such losses. Here, we compared the resistance of 10 mealworm strains to infection with the entomopathogenic bacterium *Serratia marcescens* Bizio and further assessed the effects of 2 commercial probiotics on the resistance and performance of 3 strains previously known to have wet food differential responses. Our results revealed clear strain-dependent differences in yellow mealworm growth, survival, and biomass in response to bacterial infection. While probiotic supplementation did not reduce mortality, it had variable and strain-specific effects on larval growth and yield, both when infected and under healthy conditions. These findings demonstrate that bacterial resistance in *T. molitor* is influenced by genetic background and highlight the need for strain selection in commercial farming. Probiotic use may provide benefits in certain strains, but outcomes are inconsistent, underscoring the importance of tailoring rearing practices to strain-specific responses.

## Introduction

Yellow mealworm (*Tenebrio molitor* L.) farming has expanded in recent years due to its nutritional value: mealworms are a healthy source of lipids and proteins (Mulia and Doi 2019). Notably, the yellow mealworm was the first insect approved for human consumption by the European Food and Safety Authority [EFSA Panel on Nutrition, Novel Foods and Food Allergens (NDA) et al. 2021]. Beyond direct human consumption, yellow mealworm larvae can be used as supplements in monogastric animal farming, aquaculture, as well as in medicine production and polyester degradation (Ong et al. 2018, Vigneron et al. 2019, Hong et al. 2020, Shafique et al. 2021).

However, yellow mealworm farms worldwide have also revealed risks of pathogen proliferation (Vigneron et al. 2019, Bello Gonzalez et al. 2023). Pathogen emergence is typically associated with suboptimal rearing conditions, such as excessive humidity or high rearing densities (Vigneron et al. 2019, Bello Gonzalez et al. 2023). Yellow mealworms can host a wide range of pathogens, including fungi, bacteria, viruses, nematodes, tapeworms, and other parasites (Vigneron et al. 2019). One such pathogen is the Gram-negative entomopathogen *Serratia marcensens* Bizio (Enterobacterales: Entero­bacteriaceae), which was implicated in a 30% mortality rate on a German farm (Bello Gonzalez et al. 2023).

Probiotics such as *Pediococcus pentosaceus* Mees (Lactobacillales; Lactobacillaceae) may reduce mortality in yellow mealworm farms by limiting pathogen infection or by otherwise enhancing performance (Lecocq et al. 2021, Nielsen-Leroux and Savio 2023). Two easily accessible ­probiotics, Bactocell (*Pediococcus acidilactici* Lidner, Lactobacilliales: Lactobacillaceae—Lallemand Inc., Montreal, Canada) and Levucell (*Saccharomyces cerevisiae* Meyen, Saccharomycetales: Saccharomycetaceae—Lallemand Inc., Montreal, Canada), are frequently used in agriculture. *Pediococcus acidilactici* is a Gram-positive lactic acid bacterium widely used in aquaculture and livestock, while *S. cerevisiae* has demonstrated probiotic effects across animal systems (EFSA Panel on Additives and Products or Substances used in Animal Feed (FEEDAP) 2016, 2019). Diet also plays a role: protein content in feed may enhance resistance to pathogens by upregulating antimicrobial peptide (AMP) transcription (Wilson et al. 2019). Conversely, the use of antibiotic treatments is discouraged as they may promote resistance development (Turchi et al. 2024).

Dietary protein levels influence both growth and the protein content of the mealworms themselves (Rumbos et al. 2020). Wet feed enhances feed intake but simultaneously increases the risk of pathogen outbreaks (Murray 1968, Bello Gonzalez et al. 2023, Losier 2024). In addition, recent work has revealed strain-dependent differences in yellow mealworm growth, survival and fecundity, suggesting that strain selection may critically affect the long-term revenue and success of producers (Rumbos et al. 2021, Adamaki-Sotiraki et al. 2022a,b). Yellow mealworm performance is influenced by multiple environmental factors such as temperature, humidity, feed composition, and population density, most of which can be managed by producers. Importantly, performance differences between strains can be substantial under varying environmental conditions (Ribeiro et al. 2018, Adamaki-Sotiraki et al. 2022a, Losier 2024). However, no prior study has examined whether mealworm strains differ in their resistance to bacterial infection.

Variations in bacterial resistance in *Drosophila melanogaster* Meigen (Diptera: Drosophilidae) strains was linked to genetic, geographic, and environmental effects (Lazzaro, Sackton and Clark 2006, Corby-Harris and Promislow 2008). Moreover, other tenebrionid beetles such as species of *Tribolium* (Coleoptera: Tenebrionidae) also display variations in strain-based resistance (Prendeville and Stevens 2002). Strain-specific resistance in insects is influenced by local ecosystem pressures that can drive geographic variation, and increased resistance may incur fitness costs (Dupas and Boscaro 1999, Schmid-Hempel 2005, McKean et al. 2008, Vilcinskas 2013).

Multiple factors could influence bacterial resistance variations in yellow mealworm strains. In scarce environments, yellow mealworms exhibit reduced immune function and long-term artificial selection for increased growth and biomass presents trade-offs in mortality and fecundity (Siva-Jothy and Thompson 2002, Schmid-Hempel 2005, Morales-Ramos et al. 2019). Transgenerational immune priming (TGIP) allows pathogen-exposed parents to pass increased resistance to offspring and has been documented in *T. molitor, Tribolium castaneum* (Herbst) (Coleoptera: Tenebrionidae), social insects such as *Bombus terrestris* L. (Hymenoptera: Apidae), and other invertebrates (Little et al. 2003, Moret 2006, Freitak et al. 2009, Knorr et al. 2015, Vigneron et al. 2019). While substrate bacteria trigger gut immune responses in yellow mealworm without long term effects, experiments have shown that yellow mealworm immune priming is primarily triggered by hemocoel bacterial invasion via septic wounding (Moret and Siva-Jothy 2003, Johnston et al. 2014, Dhinaut et al. 2018, Medina Gomez et al. 2018, Goerlinger et al. 2024). Therefore, rearing density of different yellow mealworm strains may drive both immune priming and TGIP, as they are likely to turn to cannibalism and injure each other when competing for feed (Maciel-Vergara et al. 2018, Barrett et al. 2023).

The objectives of this study were (i) to determine whether mealworm strains differ in their resistance to bacterial infection, and (ii) to evaluate the effects of probiotic supplementation on mealworm growth and resistance. We hypothesized that mealworm strains would display varying levels of innate resistance to bacterial infection, and that probiotic supplementation would enhance growth, yield and bacterial resistance, with strain-dependent effects.

## Materials and Methods

To test these hypotheses, we conducted 3 experiments. (i) We inoculated 10 different strains of mealworm with *S. marcescens* pellets and recorded mortality rates, growth rates and yield. (ii) We tested 2 commercial probiotics and chickpea flour on the performance of 3 mealworm strains. (iii) We evaluated the effects of the 2 probiotics on bacterial resistance in the same 3 mealworm strains.

Six of eleven strains used in this study (Greek, Italian I and II, Spanish, Turkish, and German) were generously provided by Christos G. Athanassiou, Christos Rumbos, and Christina Adamaki-Sotiraki (University of Thessaly, Greece). The 2 Norwegian strains were obtained from Invertarpro (Voss, Norway). The French strain was provided by Invers (Saint-Ignat, France), the Canadian I strain by the Worm Lady (Norwood, Canada) and the Canadian II strain by TriCycle Inc. (Montreal, Canada). Larvae from all eleven strains used in this study were submitted to the Centre de diagnostic vétérinaire de l‘Université de Montréal (CDVUM—Saint-Hyacinthe, Québec) prior to the first experiment. No pathogens were detected. All strains except for the Canadian II strain were reared at room temperature (20 °C to 25 °C) and 50% relative humidity, fed wheat bran, with carrots provided weekly as a water source. Two commercial probiotics, Bactocell (*Pediococcus acidilactici* CNCM I-4622—MA 18/5M) and Levucell (*Saccharomyces cerevisiae* CNCM I-1077) (Lallemand Inc., Montreal, Canada), were used. Probiotic concentrations were based on European food safety authority assessments (EFSA Panel on Additives and Products or Substances used in Animal Feed (FEEDAP) 2016, 2019). Treatments were standardized to ≥5% feed inclusion and ≥10^9^ CFU/g feed. The Bactocell diet contained 7.14% probiotic (10^9^ CFU/g), the Levucell diet 5% probiotic (2.05 × 10^9^ CFU/g) and the chickpea diet 20% chickpea flour. Wheat bran alone served as the control (Table 1).

**Table 1.** Treatment table for experiments 2 and 3: wheat bran (Wb) is used as a base for all treatments

	Control	Chickpea	Bactocell	Levucell
**Description**	5g Wb	4g Wb + 1 g chickpea flour	4.64g Wb + 0.36 g probiotic pellets	4.75g Wb + 0.25 g probiotic pellets
**Ratio of treatment/total feed**	NA	20%	7,14%	5%
**Mean probiotic concentration (CFU/g)**	NA	NA	1.4 × 10^10^ ([1.2:1.6] × 10^10^)	4.1 × 10^10^ ([3:6] × 10^10^)
**Calculated CFU/g of feed**	NA	NA	10^9^ ([0.9:1.1] × 10^9^)	2.05 × 10^9^ ([1.5:3] × 10^9^)
**Probiotic organism**	NA	NA	*Pediococcus acidilactici* CNCM I-4622—MA 18/5M	*Saccharomyces cerevisiae* CNCM I-1077
**Product**	NA	Chickpea flour	Bactocell PA 10	Levucell SC 20
**Provider**	NA	Shashi Foods Inc East York, Canada	Lallemand Inc. Montreal, Canada	Lallemand Inc. Montreal, Canada

Values for mean probiotic concentrations come from the EFSA FEEDAP panel.

### Experimental Design

In experiment 1, 2000 larvae from 10 strains (all but the Canadian II strain), aged 6 to 7 weeks, were selected from stock cultures (200 per strain). To minimize the effect of initial size, larvae 1.5 to 2 cm in length were chosen. As Losier (2024) previously used the Canadian II, Italian II, and Greek strains to measure the dietary effects of wet food differences, we selected these same 3 strains for our probiotic trials. To achieve identical rearing conditions, 100 adults from each of the 3 strains were divided into groups of 33 to 34 mealworms in 475 ml plastic containers with 25 g of wheat bran and agar cubes as wet food (experiment 2). After 2 days, adults were sieved out and the substrate with eggs was transferred into 3 liter plastic boxes. The process was repeated on days 4 and 7 to triple the number of starter eggs. Containers were placed in a CARON incubator (Model 6041-1; Marietta, Ohio, USA) at 27 °C and 50% to 70% relative humidity for 6 wk. After hatching, larvae were fed wheat bran and agar 3 times weekly. In experiment 3, 9 plastic boxes were filled with 500 g of feed containing control, Bactocell, or Levucell diets (Table 1). Adult oviposition and egg collection followed the same procedure described above. Larvae for experiments 2 and 3 were randomly chosen at week 6, 800 larvae from each strain for experiment 2 (2,400 in all) and 300 larvae for each strain-treatment combination for experiment 3 (2,700 in all). For all experiments, larvae were randomly separated in groups of 10 in small plastic cups used as experimental units (2.5 oz–experiment 1, 3.25 oz–experiments 2 and 3). Each combination of strain, wound (experiments 1 and 3) and feed treatment (experiments 2 and 3) were replicated 10 times (experiments 1 and 3) or 20 times (experiment 2) and their position within a block was randomized in each of 10 experimental blocks. Larvae in experiments 1 and 3 were starved prior to inoculation. Fresh agar cubes were provided to larvae as a source of water and replaced periodically. Blocks were randomly placed in 3 CARON incubator (Model 6041-1; Marietta, Ohio, USA) at 27 °C and 50%–70% relative humidity.

### Inoculation

*Serratia marcescens* spp. *marcescens* Bizio (ATCC 13380) bacterial pellets were dissolved in glycerol and stored at −80 °C. Striations on nutrient agar (Difco Laboratories, Inc., Franklin Lakes, United States) were incubated for 48 h at 26 °C. A monoclonal strain was then transferred into 40 ml of nutrient broth and incubated for another 48 h at 26 °C. Striations were repeated on 2 agar plates to confirm the absence of contamination. Ten 40 ml nutrient broth solutions were inoculated and incubated for 48 h, then centrifuged at 4,000 rpm for 10 min using an Eppendorf centrifuge (Model 5804; Hamburg, Germany). Supernatants were discarded, and the resulting pellets were resuspended in 10 ml of nutrient broth and distributed into 10 sterile 1.5 ml microcentrifuge tubes (Eppendorf, Hamburg, Germany). To inoculate larvae, we followed with slight modifications the protocol developed by Goerlinger et al. (2024), as feeding larvae with contaminated feed resulted in no mortality (unpublished data). Sterile 30G syringes (Becton Dickinson, Franklin Lakes, New Jersey, USA) were used for wounding. Experiment 1 contained 2 groups: septic wounds and sterile wounds. For septic wounds, each bacterial suspension tube was centrifuged 10 min at 4,000 rpm (MiniSpin, Eppendorf, Hamburg, Germany) and syringes were dipped in the resulting bacterial pellet prior to wounding. Sterile wounds were administered with syringes dipped in sterile nutrient broth. An additional unwounded group was added to experiment 3. This control allowed comparison of sterile wounding effects with natural larval growth. Following wounding, larvae of all treatments were isolated in 2 ml microcentrifuge tubes for 24 h to prevent healing complications due to contact with other larvae or the rearing substrate. They were then returned to their experimental units provided with 5 g of wheat bran or the corresponding feed treatment.

### Data Acquisition, Curation, and Analysis

In all experiments, surviving larvae were counted and their combined mass in each unit recorded to 4 significant figures using a SARTORIUS analytical balance (Model Practum224-1S; Göttingen, Germany). Initial combined mass was recorded on the day larvae were separated from their rearing substrate, prior to inoculation in the cases of experiments 1 and 3. Larvae were counted and their mass measured daily for 14 days in experiment 1. For experiment 2, measurements were conducted every week for 15 wk until 95% of larvae had pupated. In experiment 3, survival and combined mass were recorded on days 2, 4, 7, 9, 11, 14, 16, and 18 post-wounding. Pupae were excluded from biomass measurement but counted as alive for mortality analyses. Daily mean larval biomass was calculated by dividing the total mass by the number of surviving larvae. Growth accumulation was computed as the increase in mean biomass relative to the initial value. In experiment 1, as no larvae pupated, we combined mean biomass data with survival to evaluate yield. In experiment 3, raw biomass was used to assess yield. Data was curated in R (v. 4.4.2) using the package Tidyverse. Histograms and descriptive statistics (min, max) were generated to identify and correct aberrant data. Statistical analyses were performed with JMP Pro 18 (JMP Statistical Discovery, Cary, USA). Mortality was analyzed with repeated-measures ANOVA using the generalized linear mixed models (Poisson distribution). Fixed effects included Time, Strain, Wound and Treatment (experiments 2 and 3) plus their interactions; random effects included Block and its interactions with fixed effects. Growth and Yield data were analyzed using the same model structure. Model assumptions (homoscedasticity, normality) were verified graphically. When time significantly interacted with other factors, analyses were performed separately within each time point.

## Results

### Experiment 1: Strain Differences in Bacterial Resistance

#### Mortality

**Fig. 1.**
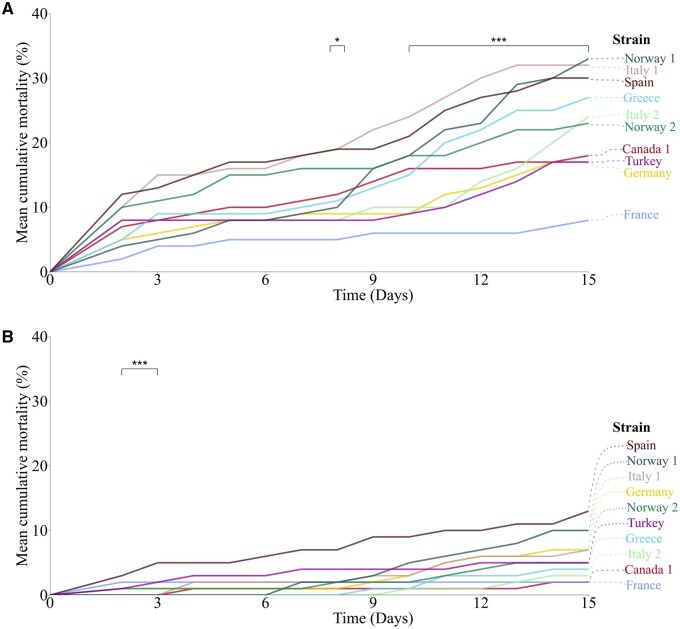
Mean cumulative mortality (%) by mealworm strains in relation to time (Days) in the first experiment. (A) Septic wounds treatment; (B) sterile wounds. Brackets indicate when mortality differences between strains were significant at *α* ≤ 0.05.

**Fig. 2.**
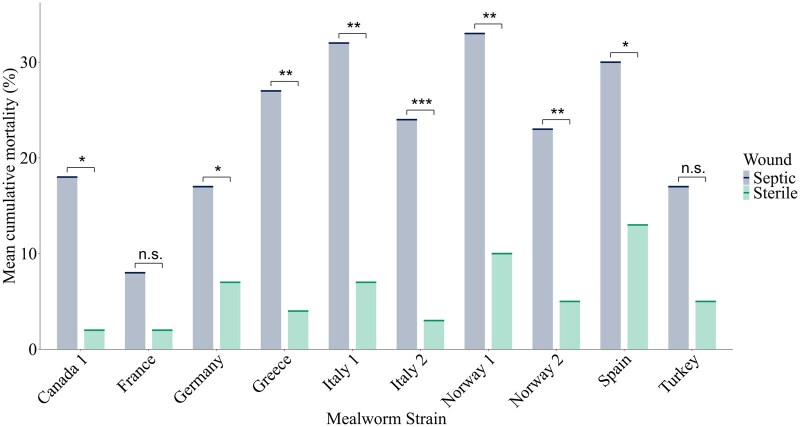
Mean cumulative mortality (%) by type of wound at the end of experiment 1 in relation to mealworm strains. (n.s.: *P* > 0.05; * *P* ≤ 0.05; ** *P* ≤ 0.01; *** *P* ≤ 0.001).

Mortality varied significantly across the interaction of wound and strain on day 2 (*F *= 3.748, df = 9,180, *P *= 0.0002) and from day 4 to the end of the experiment. For the septic wound treatment, strain differences emerged on day 8 and persisted from day 10 to the end of the trial (Fig. 1A). In the sterile wound treatment, strain differences in mortality were detected only on days 2 (*F *= 3.979, df = 9,90, *P *< 0.0001) and 3 (*F *= 4,446, df = 9,90, *P *< 0.0001), likely due to the absence of mortality in 6 of the 10 strains on those days (Fig. 1B). The French strain exhibited the lowest mortality under septic wounding (mean 8%), whereas the Spanish, Norwegian I, and Italian I strains showed the highest mortality rates (>30%). Differences between septic and sterile wounds were significant for all strains except the French (*F *= 2.211, df = 1,9.7, *P *= 0.1688) and Turkish ones (*F *= 3.912, df = 1,8.1, *P *= 0.0830; Fig. 2).

#### Growth, Mean Larval and Total Biomass (Yield)

**Fig. 3.**
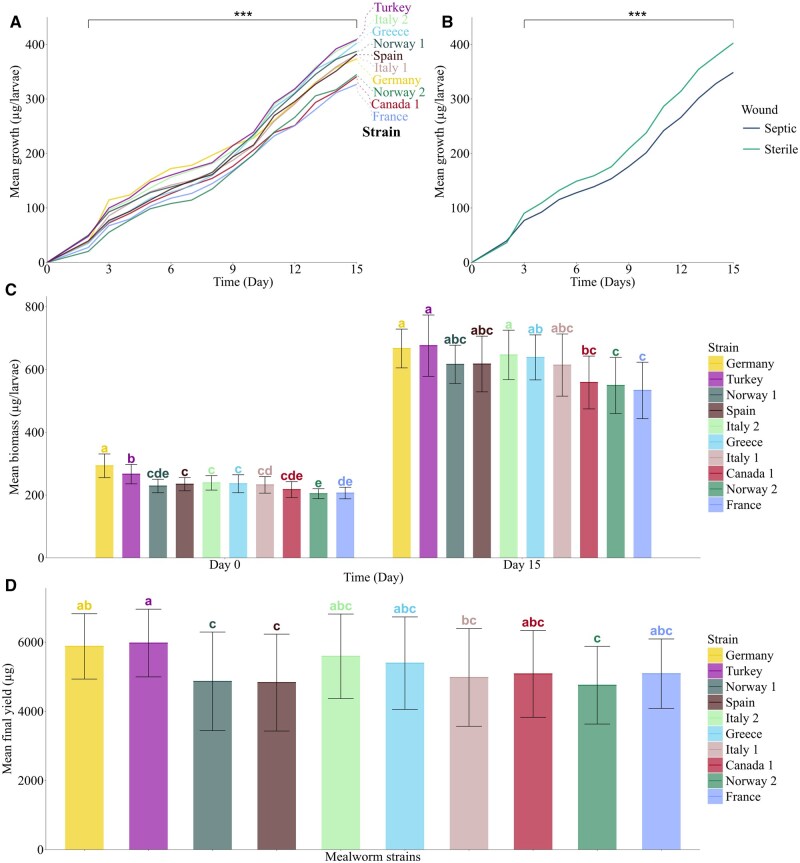
Growth, mean mass and yield results of experiment 1. Mean mealworm growth by strain (A) and wound (B) in relation to time. Brackets indicate when growth differences between wounds were significant (*** *P *≤ 0.05.) (C) Mean mealworm biomass by strain at the start (Day 0) and at the end (Day 15) of experiment 1. (D) Final mealworm yield by strain at the end of experiment 1. Statistical differences are represented by the letters on top of the bars. Strains which do not share a letter show significant differences at *α* ≤ 0.05.

Growth differed among strains from day 2 onwards (*F *= 2.554, df = 9,81, *P *= 0.0123 at the end of the experiment; Fig. 3A). Post hoc Tukey HSD tests identified differences only between the French and Turkish strains. Growth was reduced by the septic wounding from day 3 onwards (*F *= 17,93, df = 1,9, *P *= 0.0022 at the end of the experiment; Fig. 3B). Initial mean larval biomass varied among strains (*F *= 22.56, df = 9,81, *P *< 0.0001), with German and Turkish larvae being the largest, and French and Norwegian larvae the smallest (Fig. 3C). These differences persisted at the end of the experiment (*F *= 6.723, df = 9,81, *P *< 0.0001), though some strains (Greek, Italian I, Italian II, Norwegian I, and Spanish) were no longer significantly smaller than the German and Turkish strains (Fig. 3C). Septic wounds reduced mean biomass from day 8 (*F *= 8.958, df = 1,9, *P *= 0.0151) until the end of the experiment (*F *= 9.313, df = 1,9, *P *= 0.0138).

Initial total biomass reflected the same strain differences as mean biomass, since all larvae were alive at day 0 (Fig. 3C). Final total biomass differed among strains (*F *= 4.325, df = 9,81, *P *= 0.0001), although these differences did not mirror those observed in final mean biomass. Mortality influenced total biomass outcomes: all strains except the Italian I, the Norwegian I, Norwegian II, and the Spanish strains produced yields comparable to the Turkish strain (Fig. 3C and D). Total biomass was reduced by septic wounding from day 3 (*F *= 8.208, df = 1,9, *P *= 0.0186) through the end of the experiment (*F *= 17.93, df = 1,9, *P *= 0.0022).

### Experiment 2: Probiotic Effects on Mealworm Growth

#### Strain and Treatment Differences

**Fig. 4.**
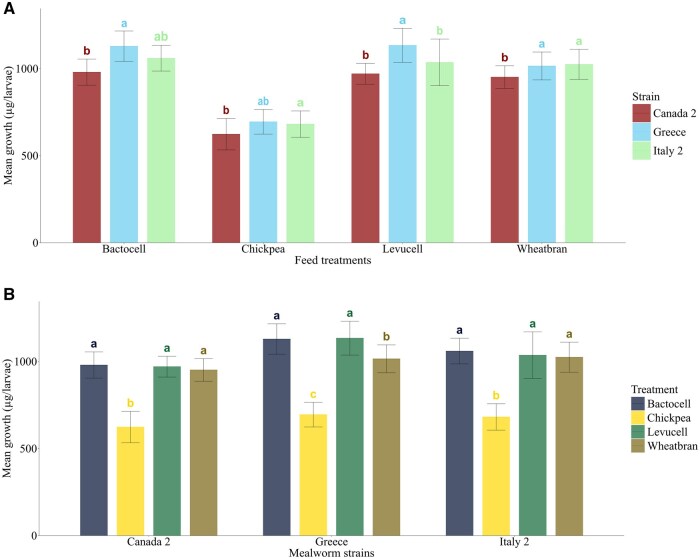
Mean mealworm growth in µg/larvae for all combinations of strains and treatments at week 10 (experiment 2). (A) Differences between treatments; (B) differences between mealworm strains. Statistical differences are represented by the letters on top of the bars. Strains which do not share a letter within a treatment or strain column show significant differences at *α* ≤ 0.05.

Initial mean larval biomass differed significantly among strains (*F *= 29.09, df = 2,222, *P *< 0.0001), with Italian II larvae being larger than those of the Canadian II and Greek strains. For growth, both strain and feed effects became significant from week 7 onwards (*F *= 33.56, df = 2,222, *P *< 0.0001; *F *= 121.8, df = 3,222, *P *< 0.0001). The interaction between strain and feed was significant at week 8 (*F *= 2.444, df = 6,222, *P *< 0.0087) and week 10 (*F *= 2.4336, df = 6,222, *P *< 0.0228). At week 10, Greek larvae showed greater growth than those of Italian II under the Levucell treatment and outperformed those of Canadian II under all diets (Fig. 4A). Canadian II and Italian II strains exhibited similar growth on Levucell and Bactocell, but the Canadian II strain performed worse under wheat bran and chickpea (Fig. 4B). Strain responses to wheat bran, Bactocell, and Levucell were consistent, although Greek larvae further benefited from both probiotics relative to wheat bran. Chickpea remained the poorest-performing diet across all strains (Fig. 4B).

### Experiment 3: No Reduction of Yellow Mealworm Mortality Under Probiotic Supplementation

#### Mortality

**Fig. 5.**
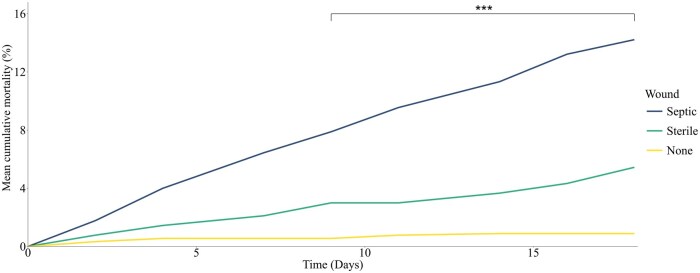
Mean cumulative mortality (%) by type of wound in relation to time for experiment 3. Brackets indicate when wound differences became significant. (***: *P* ≤ 0.001).

Mortality did not differ significantly among strains or feed treatments, but it did vary with wound type from day 9 onwards (*F *= 5.362, df = 2,35.3, *P *= 0.0093; Fig. 5). Mortality was negligible in the unwounded control (0.8%). Sterile wounds caused moderate mortality (5.4%), significantly lower than that of septic wounds (14%). Post hoc tests found no differences between unwounded and sterile wound controls.

#### Biomass Variation Across Wound, Strain, and Treatment

**Fig. 6.**
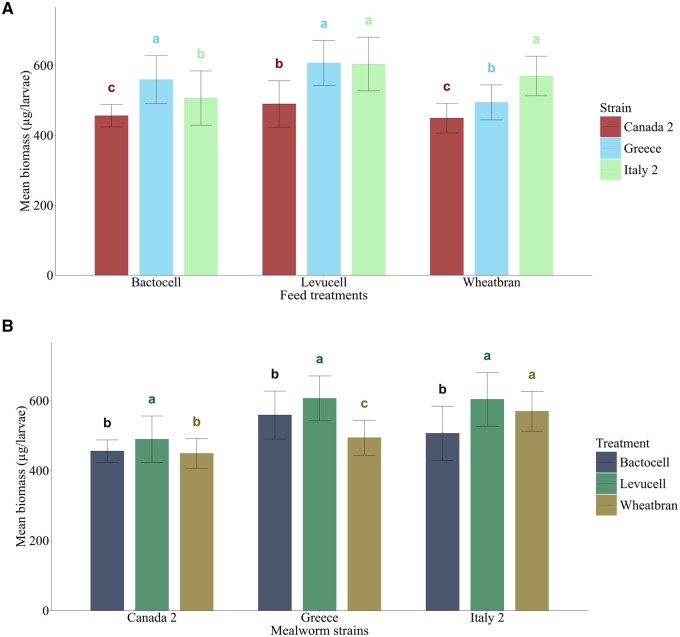
Mean initial mealworm biomass in µg/larva for all combinations of strain and treatments for experiment 3. (A) Differences between treatments; (B) differences between mealworm strains. Statistical differences are represented by the letters on top of the bars. Strains which do not share a letter within a treatment or strain column show significant differences at *α* ≤ 0.05.

Initial mean larval biomass varied with treatment (*F *= 41.46, df = 2,18, *P *< 0.0001), strain (*F *= 92.93, df = 2,18, *P *< 0.0001), and their interaction (*F *= 14.75, df = 2,18, *P *< 0.0001). Significant differences were observed within and between strains for each treatment (Fig. 6). Canadian II larvae were consistently smaller across treatments than those of the other strains. Compared to Italian II, Greek larvae were larger under Bactocell, equal under Levucell, and smaller under wheat bran (Fig. 6A). Levucell-fed larvae were larger in Canadian II and Greek strains, whereas Italian II larvae showed similar sizes as wheat bran-fed larvae, but were smaller under Bactocell. Differences between Bactocell and wheat bran varied by strain (Fig. 6B). From day 2 onwards, yield varied with strain (*F *= 137.5, df = 2,18, *P *< 0.0001), wound (*F *= 13.49, df = 2,18, *P *= 0.0003) and their interactions with treatment were all significant (*F *= 12.92, df = 4,180, *P *< 0.0001). Feed treatments were significant until day 16 (*F *= 4.371, df = 2,18, *P *< 0.0284). Three-way interactions (strain × treatment × wound) were significant at days 7 (*F *= 2.705, df = 8,180, *P *< 0.0078) and 14 (*F *= 2.395, df = 8,180, *P *< 0.0177) (Table 2). On days 7 and 14, septic wounds consistently reduced yield relative to sterile wounds and unwounded controls (Table 3). Strain-specific responses varied by diet and wound, with Greek and Italian II larvae generally outperforming Canadian larvae (Table 3). Diet effects varied, Levucell had positive or null effects on yellow mealworm growth, mean mass and yield while Bactocell showed positive results for the Greek strain, negative results for the Italian II strain and no effects on the Canadian strain (Table 3).

**Table 2.** Day-by-day ANOVA results for responses growth, mean mass, and yield by treatment (Trt), wound, strain and their interactions for experiment 3

Time	Factor	df	Response	*F*-value	*P*-value	Response	*F*-value	*P*-value	Response	*F*-value	*P*-value
**2**	Trt	2,18	Growth	3,650	0.0467	Mean	29,12	0.0001	Yield	32,55	0.0001
**2**	Wound	2,18	Growth	48,22	0.0001	Mean	7,944	0.0034	Yield	13,49	0.0003
**2**	Trt*Wound	4,180	Growth	0,7019	0.5916	Mean	0,1533	0.9613	Yield	0,2153	0.9297
**2**	Strain	2,18	Growth	17,47	0.0001	Mean	162,5	0.0001	Yield	137,5	0.0001
**2**	Trt*Strain	4,180	Growth	8,419	0.0001	Mean	13,61	0.0001	Yield	12,92	0.0001
**2**	Wound*Strain	4,180	Growth	0,9219	0.4525	Mean	0,2877	0.8857	Yield	0,3322	0.8560
**2**	Trt*Wound*Strain	8,180	Growth	1,287	0.2527	Mean	1,097	0.3670	Yield	1,125	0.3483
**4**	Trt	2,18	Growth	2,634	0.0993	Mean	20,70	0.0001	Yield	16,39	0.0001
**4**	Wound	2,18	Growth	53,31	0.0001	Mean	24,47	0.0001	Yield	51,2	0.0001
**4**	Trt*Wound	4,180	Growth	1,413	0.2316	Mean	0,8433	0.4994	Yield	0,3834	0.8203
**4**	Strain	2,18	Growth	14,10	0.0002	Mean	91,42	0.0001	Yield	54,28	0.0001
**4**	Trt*Strain	4,180	Growth	4,480	0.0018	Mean	9,773	0.0001	Yield	9,937	0.0001
**4**	Wound*Strain	4,180	Growth	1,061	0.3771	Mean	0,5503	0.6990	Yield	0,5099	0.7285
**4**	Trt*Wound*Strain	8,180	Growth	1,243	0.2763	Mean	1,353	0.2203	Yield	1,56	0.1398
**7**	Trt	2,18	Growth	0,7239	0.4984	Mean	23,50	0.0001	Yield	17,04	0.0001
**7**	Wound	2,18	Growth	144,1	0.0001	Mean	57,13	0.0001	Yield	105,4	0.0001
**7**	Trt*Wound	4,180	Growth	0,8597	0.4893	Mean	0,2978	0.8791	Yield	0,3873	0.8175
**7**	Strain	2,18	Growth	15,99	0.0001	Mean	96,61	0.0001	Yield	44,1	0.0001
**7**	Trt*Strain	4,180	Growth	4,882	0.0009	Mean	15,94	0.0001	Yield	11,09	0.0001
**7**	Wound*Strain	4,180	Growth	2,105	0.0821	Mean	0,4424	0.7778	Yield	0,1698	0.9536
**7**	Trt*Wound*Strain	8,180	Growth	2,307	0.0223	Mean	2,876	0.0049	Yield	2,705	0.0078
**9**	Trt	2,18	Growth	0,6377	0.5400	Mean	14,24	0.0002	Yield	10,46	0.0010
**9**	Wound	2,18	Growth	121,9	0.0001	Mean	63,97	0.0001	Yield	150,4	0.0001
**9**	Trt*Wound	4,180	Growth	0,2238	0.9249	Mean	0,5328	0.7118	Yield	0,1273	0.9724
**9**	Strain	2,18	Growth	29,80	0.0001	Mean	152,2	0.0001	Yield	76,81	0.0001
**9**	Trt*Strain	4,180	Growth	6,143	0.0001	Mean	12,98	0.0001	Yield	8,679	0.0001
**9**	Wound*Strain	4,180	Growth	1,498	0.2046	Mean	0,5486	0.7003	Yield	0,4529	0.7702
**9**	Trt*Wound*Strain	8,180	Growth	0,5671	0.8039	Mean	1,376	0.2099	Yield	1,056	0.3963
**11**	Trt	2,18	Growth	0,9862	0.3923	Mean	13,68	0.0002	Yield	9,235	0.0017
**11**	Wound	2,18	Growth	141,4	0.0001	Mean	126,5	0.0001	Yield	211,1	0.0001
**11**	Trt*Wound	4,180	Growth	0,1039	0.9810	Mean	0,3388	0.8515	Yield	0,1659	0.9555
**11**	Strain	2,18	Growth	30,88	0.0001	Mean	120,9	0.0001	Yield	69,51	0.0001
**11**	Trt*Strain	4,180	Growth	3,852	0.0050	Mean	9,621	0.0001	Yield	8,057	0.0001
**11**	Wound*Strain	4,180	Growth	1,505	0.2025	Mean	0,7885	0.5341	Yield	0,3257	0.8605
**11**	Trt*Wound*Strain	8,180	Growth	0,8611	0.5505	Mean	1,468	0.1716	Yield	1,562	0.1387
**14**	Trt	2,18	Growth	2,931	0.0791	Mean	17,60	0.0001	Yield	8,74	0.0022
**14**	Wound	2,18	Growth	162,8	0.0001	Mean	144,0	0.0001	Yield	207,8	0.0001
**14**	Trt*Wound	4,180	Growth	1,289	0.2759	Mean	0,3692	0.8304	Yield	0,3688	0.8306
**14**	Strain	2,18	Growth	19,89	0.0001	Mean	73,98	0.0001	Yield	33,69	0.0001
**14**	Trt*Strain	4,180	Growth	3,586	0.0077	Mean	8,102	0.0001	Yield	5,236	0.0005
**14**	Wound*Strain	4,180	Growth	1,085	0.3653	Mean	0,6519	0.6262	Yield	0,17	0.9535
**14**	Trt*Wound*Strain	8,180	Growth	1,627	0.1198	Mean	2,593	0.0105	Yield	2,39	0.0180
**16**	Trt	2,18	Growth	0,5016	0.6138	Mean	5,200	0.0165	Yield	4,371	0.0284
**16**	Wound	2,18	Growth	185,0	0.0001	Mean	151,6	0.0001	Yield	220,1	0.0001
**16**	Trt*Wound	4,180	Growth	0,5101	0.7284	Mean	0,05311	0.9947	Yield	0,2878	0.8856
**16**	Strain	2,18	Growth	17,21	0.0001	Mean	58,26	0.0001	Yield	40,04	0.0001
**16**	Trt*Strain	4,180	Growth	4,238	0.0027	Mean	8,665	0.0001	Yield	5,404	0.0004
**16**	Wound*Strain	4,180	Growth	1,290	0.2756	Mean	0,6816	0.6056	Yield	0,1444	0.9653
**16**	Trt*Wound*Strain	8,180	Growth	1,050	0.4008	Mean	1,739	0.0922	Yield	1,956	0.0544
**18**	Trt	2,18	Growth	2,518	0.1086	Mean	1,523	0.2449	Yield	1,580	0.2332
**18**	Wound	2,18	Growth	178,1	0.0001	Mean	154,1	0.0001	Yield	238,6	0.0001
**18**	Trt*Wound	4,180	Growth	0,8943	0.4686	Mean	0,2929	0.8823	Yield	0,3674	0.8317
**18**	Strain	2,18	Growth	17,31	0.0001	Mean	71,63	0.0001	Yield	37,79	0.0001
**18**	Trt*Strain	4,180	Growth	4,536	0.0016	Mean	9,386	0.0001	Yield	5,337	0.0004
**18**	Wound*Strain	4,180	Growth	2,022	0.0933	Mean	1,275	0.2813	Yield	0,2982	0.8788
**18**	Trt*Wound*Strain	8,180	Growth	1,076	0.3817	Mean	1,539	0.1466	Yield	1,612	0.1239

**Table 3.** Post hoc comparisons for 3-way interactions of strain, treatment and wound on responses growth (Day 7), mean mass and yield (Days 7 and 14; experiment 3)

Response	Days	Strain-Treatment	Wound	Letters	Strain-Wound	Feed	Letters	Treatment-Wound	Strain	Letters
**Growth**	7	Canada-Control	No_Wound	A	Canada-No_Wound	Control	A	Control-No_Wound	Canada	A
**Growth**	7	Canada-Control	Sterile	B	Canada-No_Wound	Bactocell	A	Control-No_Wound	Greece	A
**Growth**	7	Canada-Control	Septic	C	Canada-No_Wound	Levucell	A	Control-No_Wound	Italy	A
**Growth**	7	Canada-Bactocell	No_Wound	A	Canada-Sterile	Control	A	Control-Sterile	Canada	B
**Growth**	7	Canada-Bactocell	Sterile	B	Canada-Sterile	Bactocell	A	Control-Sterile	Greece	AB
**Growth**	7	Canada-Bactocell	Septic	C	Canada-Sterile	Levucell	A	Control-Sterile	Italy	A
**Growth**	7	Canada-Levucell	No_Wound	A	Canada-Septic	Control	B	Control-Septic	Canada	B
**Growth**	7	Canada-Levucell	Sterile	B	Canada-Septic	Bactocell	AB	Control-Septic	Greece	A
**Growth**	7	Canada-Levucell	Septic	C	Canada-Septic	Levucell	A	Control-Septic	Italy	A
**Growth**	7	Greece-Control	No_Wound	A	Greece-No_Wound	Control	A	Bactocell-No_Wound	Canada	A
**Growth**	7	Greece-Control	Sterile	B	Greece-No_Wound	Bactocell	A	Bactocell-No_Wound	Greece	A
**Growth**	7	Greece-Control	Septic	C	Greece-No_Wound	Levucell	A	Bactocell-No_Wound	Italy	A
**Growth**	7	Greece-Bactocell	No_Wound	A	Greece-Sterile	Control	A	Bactocell-Sterile	Canada	B
**Growth**	7	Greece-Bactocell	Sterile	A	Greece-Sterile	Bactocell	A	Bactocell-Sterile	Greece	A
**Growth**	7	Greece-Bactocell	Septic	B	Greece-Sterile	Levucell	A	Bactocell-Sterile	Italy	B
**Growth**	7	Greece-Levucell	No_Wound	A	Greece-Septic	Control	A	Bactocell-Septic	Canada	A
**Growth**	7	Greece-Levucell	Sterile	A	Greece-Septic	Bactocell	A	Bactocell-Septic	Greece	A
**Growth**	7	Greece-Levucell	Septic	B	Greece-Septic	Levucell	A	Bactocell-Septic	Italy	A
**Growth**	7	Italy-Control	No_Wound	A	Italy-No_Wound	Control	A	Levucell-No_Wound	Canada	B
**Growth**	7	Italy-Control	Sterile	A	Italy-No_Wound	Bactocell	A	Levucell-No_Wound	Greece	A
**Growth**	7	Italy-Control	Septic	B	Italy-No_Wound	Levucell	A	Levucell-No_Wound	Italy	B
**Growth**	7	Italy-Bactocell	No_Wound	A	Italy-Sterile	Control	A	Levucell-Sterile	Canada	B
**Growth**	7	Italy-Bactocell	Sterile	B	Italy-Sterile	Bactocell	B	Levucell-Sterile	Greece	A
**Growth**	7	Italy-Bactocell	Septic	C	Italy-Sterile	Levucell	A	Levucell-Sterile	Italy	AB
**Growth**	7	Italy-Levucell	No_Wound	A	Italy-Septic	Control	A	Levucell-Septic	Canada	A
**Growth**	7	Italy-Levucell	Sterile	A	Italy-Septic	Bactocell	B	Levucell-Septic	Greece	A
**Growth**	7	Italy-Levucell	Septic	B	Italy-Septic	Levucell	AB	Levucell-Septic	Italy	A
**Mass**	7	Canada-Control	No_Wound	A	Canada-No_Wound	Control	A	Control-No_Wound	Canada	B
**Mass**	7	Canada-Control	Sterile	A	Canada-No_Wound	Bactocell	A	Control-No_Wound	Greece	AB
**Mass**	7	Canada-Control	Septic	B	Canada-No_Wound	Levucell	A	Control-No_Wound	Italy	A
**Mass**	7	Canada-Bactocell	No_Wound	A	Canada-Sterile	Control	A	Control-Sterile	Canada	B
**Mass**	7	Canada-Bactocell	Sterile	B	Canada-Sterile	Bactocell	A	Control-Sterile	Greece	B
**Mass**	7	Canada-Bactocell	Septic	B	Canada-Sterile	Levucell	A	Control-Sterile	Italy	A
**Mass**	7	Canada-Levucell	No_Wound	A	Canada-Septic	Control	A	Control-Septic	Canada	C
**Mass**	7	Canada-Levucell	Sterile	AB	Canada-Septic	Bactocell	A	Control-Septic	Greece	B
**Mass**	7	Canada-Levucell	Septic	B	Canada-Septic	Levucell	A	Control-Septic	Italy	A
**Mass**	7	Greece-Control	No_Wound	A	Greece-No_Wound	Control	B	Bactocell-No_Wound	Canada	B
**Mass**	7	Greece-Control	Sterile	AB	Greece-No_Wound	Bactocell	B	Bactocell-No_Wound	Greece	A
**Mass**	7	Greece-Control	Septic	B	Greece-No_Wound	Levucell	A	Bactocell-No_Wound	Italy	AB
**Mass**	7	Greece-Bactocell	No_Wound	A	Greece-Sterile	Control	B	Bactocell-Sterile	Canada	B
**Mass**	7	Greece-Bactocell	Sterile	A	Greece-Sterile	Bactocell	A	Bactocell-Sterile	Greece	A
**Mass**	7	Greece-Bactocell	Septic	B	Greece-Sterile	Levucell	A	Bactocell-Sterile	Italy	B
**Mass**	7	Greece-Levucell	No_Wound	A	Greece-Septic	Control	B	Bactocell-Septic	Canada	B
**Mass**	7	Greece-Levucell	Sterile	B	Greece-Septic	Bactocell	AB	Bactocell-Septic	Greece	A
**Mass**	7	Greece-Levucell	Septic	B	Greece-Septic	Levucell	A	Bactocell-Septic	Italy	B
**Mass**	7	Italy-Control	No_Wound	A	Italy-No_Wound	Control	A	Levucell-No_Wound	Canada	C
**Mass**	7	Italy-Control	Sterile	AB	Italy-No_Wound	Bactocell	A	Levucell-No_Wound	Greece	A
**Mass**	7	Italy-Control	Septic	B	Italy-No_Wound	Levucell	A	Levucell-No_Wound	Italy	B
**Mass**	7	Italy-Bactocell	No_Wound	A	Italy-Sterile	Control	A	Levucell-Sterile	Canada	B
**Mass**	7	Italy-Bactocell	Sterile	AB	Italy-Sterile	Bactocell	B	Levucell-Sterile	Greece	A
**Mass**	7	Italy-Bactocell	Septic	B	Italy-Sterile	Levucell	A	Levucell-Sterile	Italy	A
**Mass**	7	Italy-Levucell	No_Wound	A	Italy-Septic	Control	A	Levucell-Septic	Canada	B
**Mass**	7	Italy-Levucell	Sterile	A	Italy-Septic	Bactocell	B	Levucell-Septic	Greece	A
**Mass**	7	Italy-Levucell	Septic	A	Italy-Septic	Levucell	A	Levucell-Septic	Italy	A
**Mass**	14	Canada-Control	No_Wound	A	Canada-No_Wound	Control	AB	Control-No_Wound	Canada	B
**Mass**	14	Canada-Control	Sterile	B	Canada-No_Wound	Bactocell	A	Control-No_Wound	Greece	A
**Mass**	14	Canada-Control	Septic	C	Canada-No_Wound	Levucell	B	Control-No_Wound	Italy	A
**Mass**	14	Canada-Bactocell	No_Wound	A	Canada-Sterile	Control	B	Control-Sterile	Canada	C
**Mass**	14	Canada-Bactocell	Sterile	A	Canada-Sterile	Bactocell	AB	Control-Sterile	Greece	B
**Mass**	14	Canada-Bactocell	Septic	B	Canada-Sterile	Levucell	A	Control-Sterile	Italy	A
**Mass**	14	Canada-Levucell	No_Wound	A	Canada-Septic	Control	A	Control-Septic	Canada	B
**Mass**	14	Canada-Levucell	Sterile	A	Canada-Septic	Bactocell	A	Control-Septic	Greece	A
**Mass**	14	Canada-Levucell	Septic	B	Canada-Septic	Levucell	A	Control-Septic	Italy	A
**Mass**	14	Greece-Control	No_Wound	A	Greece-No_Wound	Control	B	Bactocell-No_Wound	Canada	B
**Mass**	14	Greece-Control	Sterile	B	Greece-No_Wound	Bactocell	B	Bactocell-No_Wound	Greece	A
**Mass**	14	Greece-Control	Septic	B	Greece-No_Wound	Levucell	A	Bactocell-No_Wound	Italy	AB
**Mass**	14	Greece-Bactocell	No_Wound	A	Greece-Sterile	Control	A	Bactocell-Sterile	Canada	B
**Mass**	14	Greece-Bactocell	Sterile	A	Greece-Sterile	Bactocell	A	Bactocell-Sterile	Greece	A
**Mass**	14	Greece-Bactocell	Septic	B	Greece-Sterile	Levucell	A	Bactocell-Sterile	Italy	B
**Mass**	14	Greece-Levucell	No_Wound	A	Greece-Septic	Control	A	Bactocell-Septic	Canada	B
**Mass**	14	Greece-Levucell	Sterile	B	Greece-Septic	Bactocell	A	Bactocell-Septic	Greece	A
**Mass**	14	Greece-Levucell	Septic	C	Greece-Septic	Levucell	A	Bactocell-Septic	Italy	B
**Mass**	14	Italy-Control	No_Wound	A	Italy-No_Wound	Control	A	Levucell-No_Wound	Canada	B
**Mass**	14	Italy-Control	Sterile	A	Italy-No_Wound	Bactocell	A	Levucell-No_Wound	Greece	A
**Mass**	14	Italy-Control	Septic	B	Italy-No_Wound	Levucell	A	Levucell-No_Wound	Italy	B
**Mass**	14	Italy-Bactocell	No_Wound	A	Italy-Sterile	Control	A	Levucell-Sterile	Canada	B
**Mass**	14	Italy-Bactocell	Sterile	A	Italy-Sterile	Bactocell	B	Levucell-Sterile	Greece	AB
**Mass**	14	Italy-Bactocell	Septic	B	Italy-Sterile	Levucell	A	Levucell-Sterile	Italy	A
**Mass**	14	Italy-Levucell	No_Wound	A	Italy-Septic	Control	A	Levucell-Septic	Canada	B
**Mass**	14	Italy-Levucell	Sterile	A	Italy-Septic	Bactocell	B	Levucell-Septic	Greece	A
**Mass**	14	Italy-Levucell	Septic	B	Italy-Septic	Levucell	A	Levucell-Septic	Italy	A
**Yield**	7	Canada-Control	No_Wound	A	Canada-No_Wound	Control	A	Control-No_Wound	Canada	B
**Yield**	7	Canada-Control	Sterile	A	Canada-No_Wound	Bactocell	A	Control-No_Wound	Greece	AB
**Yield**	7	Canada-Control	Septic	B	Canada-No_Wound	Levucell	A	Control-No_Wound	Italy	A
**Yield**	7	Canada-Bactocell	No_Wound	A	Canada-Sterile	Control	A	Control-Sterile	Canada	B
**Yield**	7	Canada-Bactocell	Sterile	B	Canada-Sterile	Bactocell	A	Control-Sterile	Greece	B
**Yield**	7	Canada-Bactocell	Septic	C	Canada-Sterile	Levucell	A	Control-Sterile	Italy	A
**Yield**	7	Canada-Levucell	No_Wound	A	Canada-Septic	Control	A	Control-Septic	Canada	B
**Yield**	7	Canada-Levucell	Sterile	A	Canada-Septic	Bactocell	A	Control-Septic	Greece	A
**Yield**	7	Canada-Levucell	Septic	B	Canada-Septic	Levucell	A	Control-Septic	Italy	A
**Yield**	7	Greece-Control	No_Wound	A	Greece-No_Wound	Control	B	Bactocell-No_Wound	Canada	B
**Yield**	7	Greece-Control	Sterile	B	Greece-No_Wound	Bactocell	B	Bactocell-No_Wound	Greece	A
**Yield**	7	Greece-Control	Septic	B	Greece-No_Wound	Levucell	A	Bactocell-No_Wound	Italy	AB
**Yield**	7	Greece-Bactocell	No_Wound	A	Greece-Sterile	Control	B	Bactocell-Sterile	Canada	B
**Yield**	7	Greece-Bactocell	Sterile	A	Greece-Sterile	Bactocell	A	Bactocell-Sterile	Greece	A
**Yield**	7	Greece-Bactocell	Septic	B	Greece-Sterile	Levucell	A	Bactocell-Sterile	Italy	B
**Yield**	7	Greece-Levucell	No_Wound	A	Greece-Septic	Control	B	Bactocell-Septic	Canada	B
**Yield**	7	Greece-Levucell	Sterile	B	Greece-Septic	Bactocell	AB	Bactocell-Septic	Greece	A
**Yield**	7	Greece-Levucell	Septic	C	Greece-Septic	Levucell	A	Bactocell-Septic	Italy	B
**Yield**	7	Italy-Control	No_Wound	A	Italy-No_Wound	Control	A	Levucell-No_Wound	Canada	C
**Yield**	7	Italy-Control	Sterile	A	Italy-No_Wound	Bactocell	A	Levucell-No_Wound	Greece	A
**Yield**	7	Italy-Control	Septic	B	Italy-No_Wound	Levucell	A	Levucell-No_Wound	Italy	B
**Yield**	7	Italy-Bactocell	No_Wound	A	Italy-Sterile	Control	A	Levucell-Sterile	Canada	B
**Yield**	7	Italy-Bactocell	Sterile	B	Italy-Sterile	Bactocell	B	Levucell-Sterile	Greece	A
**Yield**	7	Italy-Bactocell	Septic	C	Italy-Sterile	Levucell	A	Levucell-Sterile	Italy	A
**Yield**	7	Italy-Levucell	No_Wound	A	Italy-Septic	Control	B	Levucell-Septic	Canada	B
**Yield**	7	Italy-Levucell	Sterile	A	Italy-Septic	Bactocell	C	Levucell-Septic	Greece	A
**Yield**	7	Italy-Levucell	Septic	B	Italy-Septic	Levucell	A	Levucell-Septic	Italy	A
**Yield**	14	Canada-Control	No_Wound	A	Canada-No_Wound	Control	A	Control-No_Wound	Canada	B
**Yield**	14	Canada-Control	Sterile	B	Canada-No_Wound	Bactocell	A	Control-No_Wound	Greece	A
**Yield**	14	Canada-Control	Septic	C	Canada-No_Wound	Levucell	A	Control-No_Wound	Italy	A
**Yield**	14	Canada-Bactocell	No_Wound	A	Canada-Sterile	Control	B	Control-Sterile	Canada	B
**Yield**	14	Canada-Bactocell	Sterile	B	Canada-Sterile	Bactocell	AB	Control-Sterile	Greece	AB
**Yield**	14	Canada-Bactocell	Septic	C	Canada-Sterile	Levucell	A	Control-Sterile	Italy	A
**Yield**	14	Canada-Levucell	No_Wound	A	Canada-Septic	Control	A	Control-Septic	Canada	B
**Yield**	14	Canada-Levucell	Sterile	A	Canada-Septic	Bactocell	A	Control-Septic	Greece	A
**Yield**	14	Canada-Levucell	Septic	B	Canada-Septic	Levucell	A	Control-Septic	Italy	A
**Yield**	14	Greece-Control	No_Wound	A	Greece-No_Wound	Control	AB	Bactocell-No_Wound	Canada	A
**Yield**	14	Greece-Control	Sterile	B	Greece-No_Wound	Bactocell	B	Bactocell-No_Wound	Greece	A
**Yield**	14	Greece-Control	Septic	C	Greece-No_Wound	Levucell	A	Bactocell-No_Wound	Italy	A
**Yield**	14	Greece-Bactocell	No_Wound	A	Greece-Sterile	Control	A	Bactocell-Sterile	Canada	B
**Yield**	14	Greece-Bactocell	Sterile	A	Greece-Sterile	Bactocell	A	Bactocell-Sterile	Greece	A
**Yield**	14	Greece-Bactocell	Septic	B	Greece-Sterile	Levucell	A	Bactocell-Sterile	Italy	B
**Yield**	14	Greece-Levucell	No_Wound	A	Greece-Septic	Control	A	Bactocell-Septic	Canada	B
**Yield**	14	Greece-Levucell	Sterile	B	Greece-Septic	Bactocell	A	Bactocell-Septic	Greece	A
**Yield**	14	Greece-Levucell	Septic	C	Greece-Septic	Levucell	A	Bactocell-Septic	Italy	B
**Yield**	14	Italy-Control	No_Wound	A	Italy-No_Wound	Control	A	Levucell-No_Wound	Canada	B
**Yield**	14	Italy-Control	Sterile	A	Italy-No_Wound	Bactocell	A	Levucell-No_Wound	Greece	A
**Yield**	14	Italy-Control	Septic	B	Italy-No_Wound	Levucell	A	Levucell-No_Wound	Italy	B
**Yield**	14	Italy-Bactocell	No_Wound	A	Italy-Sterile	Control	AB	Levucell-Sterile	Canada	A
**Yield**	14	Italy-Bactocell	Sterile	B	Italy-Sterile	Bactocell	B	Levucell-Sterile	Greece	A
**Yield**	14	Italy-Bactocell	Septic	C	Italy-Sterile	Levucell	A	Levucell-Sterile	Italy	A
**Yield**	14	Italy-Levucell	No_Wound	A	Italy-Septic	Control	AB	Levucell-Septic	Canada	B
**Yield**	14	Italy-Levucell	Sterile	A	Italy-Septic	Bactocell	B	Levucell-Septic	Greece	AB
**Yield**	14	Italy-Levucell	Septic	B	Italy-Septic	Levucell	A	Levucell-Septic	Italy	A

Different letters indicate significant differences at *α* ≤ 0.05, with A > B > C. Groups sharing a letter are not significantly different (A = AB; AB = B).

## Discussion

Our first experiment confirmed that yellow mealworm strains differ in their resistance to bacterial infection. Larval performance traits–growth, survival, feed intake, harvest biomass, and fecundity–are central to farm revenues (Rumbos et al. 2021). In our study, some strains performed consistently better than others. The Turkish strain, for instance, exhibited low mortality and the highest mean mass at the end of the experiment. This is partly explained by its higher starting mass, although it has also been reported to perform best under drought conditions (Adamaki-Sotiraki et al. 2022a). Interestingly, the French strain, which initially appeared poor in terms of growth and mass, displayed the lowest mortality. Ultimately, its accumulated biomass matched other strains, suggesting that minimizing mortality can be as important as maximizing growth or harvest weight. This aligns with findings by Morales-Ramos et al. (2019), who showed that artificial selection for growth and fecundity increases mortality. We hypothesize that heritable traits associated with rapid growth may also reduce pathogen resistance, as strains with higher growth (Greek, Italian I, Norwegian I, and Spanish) experienced higher mortality following septic wounding. In contrast, the Canadian I and Norwegian II strains performed poorly, with growth and biomass comparable to the French strain but without its survival advantage. TGIP of adults may have played a role: while it reduces larval mortality, it also slows growth due to metabolic costs, potentially explaining the French strain’s pattern of low mortality but reduced growth (Vigneron et al. 2019). Pathogen resistance is also influenced by environmental factors. Feed additives can stimulate immune pathways: AMPs have been proposed as alternatives to antibiotics, as they provide broad protection against pathogens accumulating in frass (Vigneron et al. 2019, Syahrulawal et al. 2023). Genetic background is another key determinant: hybrid strains often outperform inbred ones and inbreeding can weaken immunity (Rantala et al. 2011, Adamaki-Sotiraki et al. 2023a,b). Genetic diversity may thus enhance bacterial resistance.

Based on previous observations of improved mealworm performance driven by probiotics, positive effects on all strains were expected. However, in the second experiment, only the Greek strain benefited from probiotic supplementation. Similar variable strain response patterns under varying wet feed supplementation were previously discussed for the same 3 strains: Greek larvae displayed variability, Italian II larvae remained stable and Canadian II larvae performed worst (Losier 2024). Related microorganisms, such as *Pediococcus pentosaseus*, can improve mealworm performance, and brewer’s yeast or spent grain have similarly enhanced growth and survival (Kim et al. 2019, Lecocq et al. 2021, Mancini et al. 2022). *Pediococcus pentosaceus* inhibits pathogens such as *Bacillus thuringiensis* (Bacilliales: Bacillaceae), *Pseudomonas aeruginosa* (Pseudo­monadales: Pseudomonadaceae) and *S. marcensens*, colonizes the mealworm gut, alters microbial communities, and improves growth (Lecocq et al. 2021). Probiotics have also reduced mortality from *Metarhizium brunneum* and increased antifungal peptides transcription following *Beauveria bassania* (Hypocreales: Cordycipitaceae) challenge (Dahal et al. 2022, Candian and Tedeschi 2023). If present, these effects were variable in our third experiment. The Canadian II strain did not benefit from any treatment. Levucell improved yield in Greek and Italian II strains following *S. marcensens* infection, whereas Bactocell reduced yield in Italian II under both sterile and septic wounding. Microbiome differences may underlie these variable responses. Insects such as *Anopheles* sp. (Diptera: Culicidae)*, Apis mellifera* L. (Hymenoptera: Apidae) and *Tribolium castaneum* harbor strain-specific microbiomes (Yan and Stevens 1994, Ellegaard et al. 2020, Singh et al. 2022). In mealworms, microbiome composition shifts when rearing conditions are altered and can buffer nutritional stress ( Khanal et al. 2023). DNA metabarcoding of mealworm gut contents could help clarify strain-dependent responses to probiotics (Shelomi and Chen 2020). Yield under sterile wounding was often lower than in unwounded controls, suggesting that even minor injuries may alter larval performance. This effect varied by strain and treatment, underscoring the complexity of host–environment–microbiome interactions. Given the costs of probiotics, their limited efficacy in some strains has practical implications for production. Future studies should integrate microbiome profiling and antimicrobial activity assays to strengthen interpretation. Moreover, the probiotics tested here were developed for pigs, poultry, fish, and shrimp, and may not be well adapted to insects. Insect-specific probiotics may yield better results.

The lack of strain differences and general low mortality in the third experiment was unexpected. Based on the first experiment, Greek and Italian II strains were expected to reach >20% mortality, and the Canadian II up to 30% (unpublished data). The reduced mortality may be due to improved rearing conditions, as larvae in the first experiment were maintained at room temperature in stock colonies, whereas those in the third experiment were incubated under controlled conditions. Another factor may be handling frequency: larvae in the first experiment were weighed daily for 2 wk, while in the third experiment they were disturbed only 8 times over 3 wk. Increased handling may have elevated stress and mortality in earlier trials. Despite this reduction, septic wounds still resulted in higher mortality than either control. Overall, our results support the hypothesis that mealworm strains differ in bacterial resistance and probiotic responsiveness. Importantly, the interaction between strain and treatment suggests that farm outcomes may vary depending on the genetic background or life history of the colony. While Levucell improved bacterial resistance in some strains, it had limited effects on healthy larvae, reducing its economic appeal. Bactocell, by contrast, showed negative or negligible effects and appears unsuitable for mealworm production.
